# Importance of *Candida* Antigenic Factors: Structure-Driven Immunomodulation Properties of Synthetically Prepared Mannooligosaccharides in RAW264.7 Macrophages

**DOI:** 10.3389/fcimb.2019.00378

**Published:** 2019-11-08

**Authors:** Ema Paulovičová, Lucia Paulovičová, Pavol Farkaš, Alexander A. Karelin, Yury E. Tsvetkov, Vadim B. Krylov, Nikolay E. Nifantiev

**Affiliations:** ^1^Cell Culture & Immunology Laboratory, Department of Immunochemistry of Glycoconjugates, Center for Glycomics, Institute of Chemistry, Slovak Academy of Sciences, Bratislava, Slovakia; ^2^Laboratory of Glycoconjugate Chemistry, N.D. Zelinsky Institute of Organic Chemistry, Russian Academy of Sciences, Moscow, Russia

**Keywords:** *Candida*, oligomannosides, RAW 264.7, cytokines, proliferation

## Abstract

The incidence and prevalence of serious fungal infections is rising, especially in immunosuppressed individuals. Moreover, co-administration of antibiotics and immunosuppressants has driven the emergence of new multidrug-resistant pathogens. The significant increase of multidrug-resistant pathogens, together with their ability to form biofilms, is associated with morbidity and mortality. Research on novel synthetically prepared immunomodulators as potential antifungal immunotherapeutics is of serious interest. Our study demonstrated the immunobiological activity of synthetically prepared biotinylated mannooligosaccharides mimicking *Candida* antigenic factors using RAW264.7 macrophages. Macrophage exposure to the set of eight structurally different mannooligosaccharides induced a release of Th1, Th2, Th17, and Treg cytokine signature patterns. The observed immune responses were tightly associated with structure, dose, exposure time, and selected signature cytokines. The viability/cytotoxicity of the mannooligosaccharide formulas was assessed based on cell proliferation. The structure-based immunomodulatory activity of the formulas was evaluated with respect to the length, branching and conformation of the various formulas. Glycoconjugate formulas with terminal β-mannosyl-units tended to be more potent in terms of *Candida* relevant cytokines IL-12 p70, IL-17, GM-CSF, IL-6, and TNFα induction and cell proliferation, and this tendency was associated with structural differences between the studied glycoconjugate formulas. The eight tested mannooligosaccharide conjugates can be considered potential *in vitro* immunomodulative agents suitable for *in vitro Candida* diagnostics or prospectively for subcellular anti-*Candida* vaccine design.

## Introduction

Most *Candida* species, including the facultative pathogenic strains, belong to the normal commensal mycobiota of immunocompetent individuals. The factors affecting the candidosis are diverse, including the prolonged antifungal treatment in long-term care, immunosuppression associated with anticancer therapy and transplantation of solid organ or bone marrow, immunosuppressive states as diabetes mellitus and HIV, use of vascular devices and hospitalization at intensive care units (Richter et al., 2005; Angiolella et al., 2008; Adiguzel et al., 2010; Cortés and Corrales, 2018). Next, immunocompromised persons with genetic immune system defects are at high risk for mucocutaneous and invasive fungal infections (Vinh, 2011; Cunha and Carvalho, 2012; Pichard et al., 2015; Beenhouwer, 2018). Approximately 17 different *Candida* species are known etiological agents of human infections; more than 90% of systemic infections are caused by *Candida albicans (C. albicans), Candida glabrata (C. glabrata), Candida parapsilosis (C. parapsilosis), Candida tropicalis (C. tropicalis)*, and *Candida krusei (C. krusei)* (Pfaller et al., 2002). The new multidrug-resistant species *Candida auris (C. auris)* was recently isolated (Sears and Schwartz, 2017; Forsberg et al., 2019). CD4^+^-derived T-cell subpopulations Th1, Th2, and Th17 contribute to anti-*Candida* cellular immune protection. The protective anticandidal Th1 response requires the activity of various cytokines, such as interferon gamma (IFN-γ), transforming growth factor beta (TGF-β), interleukin 6 (IL-6), tumor necrosis factor alpha (TNFα), and IL-12. The induction of the protective antifungal Th1 immune response is inhibited by Th2 cytokines, such as IL-4 and IL-10 (Ito, 2011; Netea et al., 2015; Richardson and Moyes, 2015; Gow et al., 2017). In early infection, neutralization of Th1 cytokines, mainly IFN-γ and IL-12, leads predominately to the onset of Th2 rather than Th1 responses. Th2-type responses are frequently associated with susceptibility to recurrent or persistent infection and fungal allergy. TNFα, IL-1β, IL-6, IL-8, and colony-stimulating factors (CSFs) are among the major proinflammatory cytokines associated with the interaction of immune-competent cells with *Candida* cells. TNFα is thought to be essential in the primary control of disseminated infection caused by *C. albicans*. Although IL-1 shares common properties with TNFα, both IL-1β and IL-6, acting mainly through recruitment of polymorphonuclear neutrophils (PMNs), presumably are not as essential as TNFα in the innate antifungal response. IL-12 is recognized as essential to induce the protective Th1 response to the fungus, simultaneously blocking the Th2 response. The crucial role of the Th17 subset has been associated with anti-*Candida* effectiveness, especially the mucosal immune response (Romani, 2003; Rizzetto et al., 2010; van de Veerdonk and Netea, 2010). Proinflammatory cytokines, such as IL-12, IL-15, and TNFα, have been studied as candidate adjuvants in preclinical trials based on their ability to upregulate the antifungal Th1 response (Ashman and Papadimitriou, 1995; Romani, 2011; Pikman and Ben-Ami, 2012; Naglik, 2014).

Fungal cell wall antigenically active polysaccharides, such as N-linked and O-linked α- and β-mannans, chitin, α- and β-glucans, galactomannan, galactosaminogalactan, glucuronoxylomannan, and some others, are essential immunogens that play crucial roles during host-fungus interactive communication. Cell-wall components act as pathogen-associated molecular patterns (PAMPs), recognized by the immune system through pattern recognition receptors (PRRs) such as TLR2, TLR4, dectin-2, dectin-1, Mincle, DC-SIGN, or galectin-3, on the surfaces of epithelia and myeloid cells (Netea et al., 2006, 2008, 2015; Moyes and Naglik, 2011; Perez-Garcia et al., 2011; Romani, 2011; Cunha and Carvalho, 2012; Salek-Ardakani et al., 2012; Hall and Gow, 2013; Moyes et al., 2015; Zheng et al., 2015; Gow et al., 2017; Snarr et al., 2017).

Generally, specific PAMP–PRR interactions activate the inflammatory response by triggering interleukins and growth factors cell release and phagocytosis. *O*-linked mannans are recognized via TL4 receptor (Netea et al., 2006), α- linked *N*- mannans are sensed through mannose receptor, dectin-2, Mincle, and DC-SIGN (Harris et al., 2009; McKenzie et al., 2010), and the specific receptor for β-mannan is galectin-3 (Jouault et al., 2006; Linden et al., 2013). Chitin cooperates with the mannose receptor and induces TLR9 and NOD-2dependentIL-10 release (Wagener et al., 2014; Erwig and Gow, 2016). Recently, it has been demonstrated that chitin particles of small size stimulated IL-17, IL-12, IL-23, IL-10, and TNF-α in macrophages via a MyD88- and TLR2-dependent pathway (Da Silva et al., 2008, 2009). Additionally, Dectin-1 receptor on macrophages and TLR-2 recognizes β-1,3-glucan (Brown and Gordon, 2001; Brown et al., 2002, 2003; Brown, 2006). Dectin-1 uses Syk kinase and the CARD9 to stimulate IL-10, TLR2 via the MyD88 is required for the production of IL-12p40 (Dennehy et al., 2008; Netea et al., 2008), and both pathways collaborate in TNF- stimulation. Moreover, dectin-1 and galectin-3 interact synergistically to improve the outcome of host immune response to *C. albicans* (Gantner et al., 2003; Taylor et al., 2007; Esteban et al., 2011).

The antigenic factors of mannan from medically relevant *Candida* species have been characterized and their chemical structures determined in several studies (Nishikawa et al., 1982; Suzuki and Fukazawa, 1982; Shibata et al., 1995; Fukazawa et al., 1997; Suzuki, 1997). The antigenic determinants of cell wall polysaccharides and oligosaccharides from medically important yeasts have been studied for their serological specificity and biological activity (Fukazawa et al., 1997). The investigation of species-specific antigenic factor variations of *Candida* mannan and oligomannosyl structures is essential to evaluate the structure-activity relationship, since mannan structure and epitope availability intensely affect its immunobiological behavior (Trinel et al., 1992; Fukazawa et al., 1997; Suzuki, 1997; Shibata et al., 2007). The particular structure of mannan, comprising an α-1,6-mannoside backbone and side chains with α/β-1,2-mannoside or α/β-1,3-mannoside moieties of variable lengths, varies for different *Candida* species and is dependent on the expression of a complex network of mannan biosynthesis, trafficking, and cell wall remodeling genes (Shibata et al., 2012). Different growth conditions are likely to modulate the activation of cell wall signaling cascades, expression of cell wall biosynthesis genes, and alterations in mannan composition (Ernst and Pla, 2011; Lowman et al., 2011). The role of mannosylation in fungal biology and virulence has been studied using *C. albicans* mutants; the suitability of these mutants for exploring the significance of specific mannan epitopes on cell function, pathogenesis and immune recognition has been proposed (Hall and Gow, 2013; Hall et al., 2013; West et al., 2013). Several studies have attempted to design and develop an anti-*Candida* vaccine based on cell wall-derived structures (Ito, 2011; Richardson and Moyes, 2015; Tso et al., 2018; Piccione et al., 2019). The immunogenic polysaccharide cell wall structures applied in experimental vaccine models include 65 kDa mannoproteins (Sandini et al., 2007), β-1,3-glucan (Torosantucci et al., 2005), and β-1,2- mannosides (Han et al., 1999; Cutler, 2005). These model structures were effective in humoral antibody-mediated antifungal protection. Several monoclonal antibodies were protective in preclinical studies: anti- β-1,3-glucan mAb2G8 (Torosantucci et al., 2005), anti-mannoprotein mAb C7 (Moragues et al., 2003), anti-idiotypic antibodies (Magliani et al., 2004), anti-mannan mAb (Han et al., 1999; Cutler, 2005), and anti-glycosyl mAb (Kavishwar and Shukla, 2006). These antibodies efficiently appeared as candidacidal (Moragues et al., 2003; Magliani et al., 2004; Kavishwar and Shukla, 2006), growth inhibitory, or they neutralized heat shock protein 90 (Hsp90) (Torosantucci et al., 2005). Moreover, mannan conjugated in certain vaccine formulas has already been included in clinical trials (Apostolopoulos et al., 2006; Pashov et al., 2011).

Mannan has also been studied as a promising bioactive material for drug nanocarrier systems and vaccine adjuvant formulations (Tang et al., 2009). Moreover, nanoliposomes with orthogonally bound mannan represent a platform for the development of targeted drug delivery systems and self-adjuvanted carriers for construction of recombinant vaccines (Bartheldyova et al., 2019). Concerning the design of anti-fungal vaccination therapy, apart from *Candida* cell wall moieties, potential new anti-*Candida* drugs have targeted the growth and virulence factors of *C. albicans*, including core signaling components of the high-osmolarity glycerol (HOG) and target of rapamycin (TOR) signaling pathways (Li et al., 2015), as well as various immunomodulators, e.g., colony-stimulating factors and proinflammatory cytokines (Pikman and Ben-Ami, 2012).

**Figure 1:**
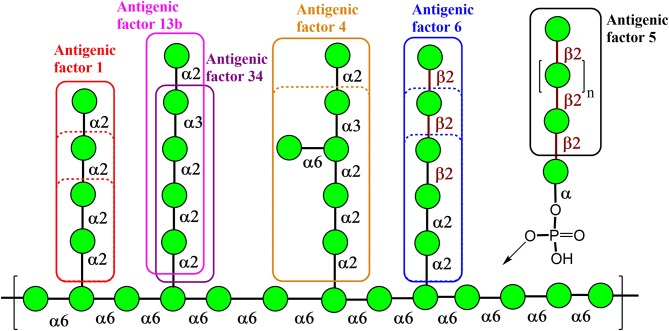
Key structural elements of the cell wall mannan from the fungus *Candida albicans* representing its antigenic factors. The carbohydrate sequences are represented according to symbol carbohydrate nomenclature (Varki et al., 1999).

Natural *Candida* mannan is a complex polysaccharide structure containing linear and branched fragments composed of α- and β-mannose units, as seen in Figure 1 (Klis et al., 2001), with the carbohydrate sequences represented according to symbol carbohydrate nomenclature (Varki et al., 1999). Thus, the use of such a heterogenic structure is problematic for the assessment of the biological role of its distinct fragments. However, the application of synthetic mannooligosaccharide derivatives, related to the structures of selected antigenic factors of *Candida* mannan, creates the opportunity to assess the biological roles of each antigenic factor.

Our work investigated the immunomodulation properties of antigenically active distinct parts of *C. albicans* mannan by using a series of structurally related synthetic mannooligosaccharides.

## Materials and Methods

### Synthesis of Biotinylated Oligomannosides 1–8

**Figure 2:**
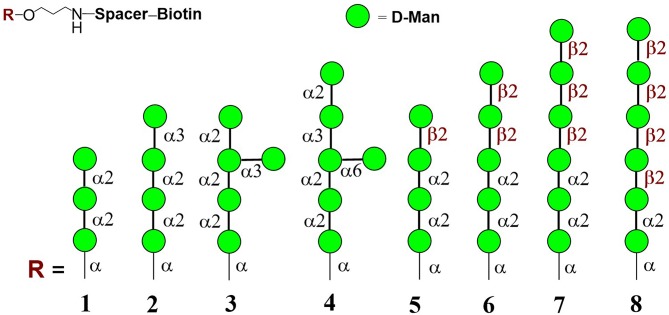
Structures of studied biotinylated mannooligosaccharides **1**–**8** (Argunov et al., 2019).

Mannooligosaccharide conjugate formulas **1** (Krylov et al., 2018a), **2** (Krylov et al., 2018a,b), **3** (Karelin et al., 2007), **4** (Karelin et al., 2010), and **5–8** (Karelin et al., 2016) were prepared by the biotinylation of parent ligands according to previously described biotinylation protocols (Figure 2) (Tsvetkov et al., 2012).

### Isolation of Natural Cellular Mannan and Preparation of FITC-Labeled Mannan

The yeast strain *Candida albicans* CCY 29-3-100 (serotype A) (CCY Culture Collection of Yeasts, Institute of Chemistry, Center for Glycomics, Slovak Academy of Sciences, Bratislava, Slovakia) was used to isolate and purify cellular mannan from fresh biomass. Mannan was extracted by autoclaving in 0.2 mol/l NaCl (120°C, 700 kPa) for 10 min and purified using precipitation with Fehling reagent according to a previously described method (Peat et al., 1961).

For the preparation of FITC-labeled mannan, *C. albicans* CCY 29-3-100 mannan (54 mg) was suspended in 1.00 mL of dimethyl sulfoxide and 2.0 μL of pyridine was added. The suspension was heated in a thermoblock at 95°C until the mannan dissolved (3 h). Then, 20 mg of isothiocyanatofluorescein (FITC) was added and heated for another 2 h at 95°C. The reaction was quenched by addition of 10 mL of water, and the result was dialyzed using cellulose membrane tube (cut-off = 14,000, Sigma) against 0.05 % NaHCO3 (1 × 0.9 L, 4 h stirred) and deionized water [8 × 0.9 L, 4 h on stirrer or 12 h in the refrigerator (5°C)] and then lyophilized (FreeZone 18 Liter Console Freeze Dry System, Labconco Corporation, Kansas City, USA).

### Preparation of Stock Solutions of Natural Cellular Mannan and Synthetically Prepared Mannooligosaccharides

Stock solutions and different dilutions of natural cellular mannan and glycoconjugate formulas **1–8** were prepared aseptically using pre-sterilized disposable plastic wares and sterile, apyrogenic aqua pro injectione (Fresenius Kabi Italia S.r.l., Verona, Italy). All solutions were prepared in a laminar flow hood and sterilized using a 0.2-μm filter (Q-Max®Syringe filter, Frisenette ApS, Knebel, Denmark) before exposure. The laminar flow cabinet was sterilized with 70% ethanol p.a. and UV for 30 min prior to each experiment. The stock solutions were assayed with EndoLISA® ELISA-based Endotoxin Detection Assay (Hyglos, Bernried am Starnberger See, Germany) and evaluated using the Cytation 5 Imager Multi-Mode Reader (BioTek, Winooski, USA) to ascertain endotoxin-free exposure conditions.

### Cell Maintenance and Culture, Cell Exposure

The murine macrophage-like RAW 264.7 cell line was selected in the present study because this cell model has been frequently used in *in vitro* studies on phagocytosis, cytokine production, and to evaluate potential bioactive substances to predict their effect *in vivo*.

RAW 264.7 (ATCC®TIB-71^TM^, ATCC, Manassas, USA) cells were cultured in complete Dulbecco's Modified Eagle Medium for 24 h and 48 h, at 37°C under 5% CO_2_ atmosphere and 90–100% relative humidity until ~80% confluence. Viability of cells was determined by Trypan Blue dye exclusion method using a TC20^TM^ automated cell counter (Bio-Rad Laboratories, Inc., Hercules, USA). The starting inoculum of 1 × 10^5^ cells/mL/well (98.3% of viable cells) was seeded in a 24-well cell culture plate (Sigma-Aldrich, St. Louis USA) and exposed to 10 and 100 μg per well of glycoconjugates for 24 and 48 h. Cell mitogens Concanavalin A (Con A; 10 μg/mL, Sigma-Aldrich), phytohemagglutinin (PHA; 10 μg/mL, Sigma-Aldrich), pokeweed mitogen (PWM, 1 μg/mL, Sigma-Aldrich), and lipopolysaccharide (LPS; 1 μg/mL, Sigma-Aldrich) were used as positive controls. The cell culture media were separated and stored at −20**°**C until further use. Cell morphology and viability were assayed before ELISA and evaluation of cytotoxicity. The interaction of FITC-labeled *Candida* mannan (100 μg/mL) and RAW 264.7 macrophage cells (1 × 10^5^ cells/mL) was evaluated using either light and fluorescence microscopy (AxioVision Imager A.1, magnification 630x; Zeiss, Wetzlar, Germany) or confocal imaging (Axio Observer LSM 880 employing an Airyscan Plan-Apochromat 63x/1.4 oil DIC M27 optical lens and Zen 2 software) with application of 3D Z-stack imaging (Zeiss).

### Cell Proliferation and Cytotoxicity

The influence of glycoconjugates on RAW 264.7 cell proliferation and cytotoxicity was evaluated using the cell proliferation assay ViaLight^TM^ plus kit (Lonza, Rockland, ME, USA) according to the manufacturer's recommendations. Cellular ATP was determined with luciferase-based luminescence quantification. The intensity of emitted light was measured using the Cytation 5 Cell Imaging Multi-Mode Reader (BioTek Instruments, Inc.). Light emission was recorded continuously for 1 s and peak values were evaluated and expressed as relative light units (RLU). The values of unexposed cells were considered the baseline. The proliferation index was calculated as the ratio between the stimulated cells (glycoconjugate formula-treated cells) and the baseline proliferation of unexposed cells. Thus, the proliferation index of the negative control, i.e., unexposed cells, was equal to one.

### Determination of Interleukins and Growth Factors

The levels of interleukins and growth factors in cell culture supernates induced by exposure with glycoconjugate formulas **1**–**8** were assayed according to the manufacturer's instructions with Platinum ELISAs® (eBioscience, Thermo Fisher Scientific, Waltham, USA): Mouse IL-12 p70 (MDD 4 pg/mL), Mouse granulocyte-macrophage colony-stimulating factor (GM-CSF;MDD 2 pg/mL), Mouse IL-17 (MDD 1.6 pg/mL), and Mouse IL-6 (MDD 6.5 pg/mL), and Instant ELISAs® (eBioscience): Mouse tumor necrosis factor (TNF)-α (MDD 4 pg/mL), and Mouse IL-10 (MDD 5.28 pg/mL).

To compare the effect of different glycoconjugates on RAW 264.7 macrophage interleukins and growth factors, analyses were performed on raw cytokine concentration data and cytokine concentration data normalized to viable cell counts of untreated control RAW 264.7 cells. The raw concentrations of cytokines determined by ELISA were divided by the RLU [ATP detection systems to quantify viable cells, ViaLightTM plus kit (Lonza, USA)] of living cells in a corresponding sample and multiplied by the RLU of untreated control RAW 264.7 cells.

### Statistical Analysis

The experimental results were expressed as mean values ± SD. Normality of data distribution was established according to the Shapiro–Wilk test at the 0.05 level of significance. Statistical comparisons were performed by one-way ANOVA and *post-hoc* Bonferroni tests. Pearson's correlation coefficient was used to compare the strength of the relationship between immunobiological variables. Results were significant when the differences equaled or exceeded the 95% confidence level (*P* < 0.05). Statistics were performed using ORIGIN 7.5 PRO software (OriginLab Corporation, Northampton, USA).

## Results

Modern chemical methods enable regio- and stereoselective assembling of linear and branched structures similar to *C. albicans* mannan (Collot et al., 2009; Karelin et al., 2017; Krylov et al., 2017). Here we report the results of our investigation into the structure-driven immunomodulating properties of synthetically prepared mannooligosaccharides in RAW264.7 macrophages using a synthetically prepared panel of biotinylated mannooligosaccharides, formulas **1**–**8** (Figure 2). These oligomannosides represented antigenic factor 1 (formula **1**), factor 34 (formula **2**), factor 4 (formula **4**), and factor 6 (formulas **5**–**8**) of *C. albicans* mannan. Side chains related to formula **3** were also found in *C. albicans* mannan (Kogan et al., 1988), but their antigenic specificity is not yet clear.

**Figure 3:**
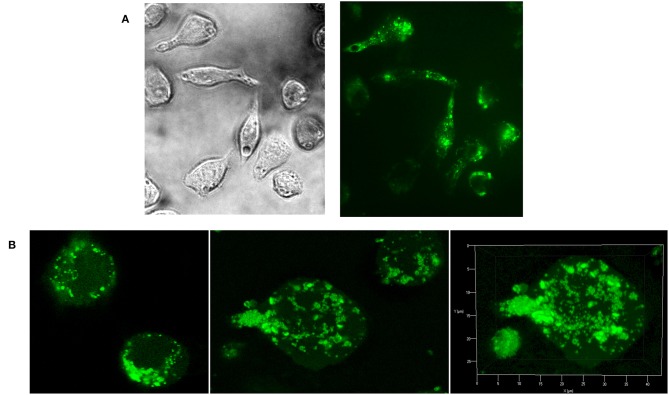
Evaluation of Raw 264.7 cellular interactions with *C. albicans* mannan- FITC conjugated complex. **(A)** light and fluorescence microscopy (magnification 630x); **(B)** confocal microscopy.

As natural *Candida* mannan (section Isolation of natural cellular mannan and preparation of FITC-labeled mannan) was utilized in all experiments as a comparative substance, evaluation of its interaction with RAW 264.7 cells was essential. Fluorescently labeled natural mannan was used to visualize the cell interaction and endocytotosis of *Candida* mannan by the murine macrophage RAW 264.7 cells. Evaluation of the interaction was performed with light and fluorescence microscopy (Figure 3A) and 3D Z-stack imaging (Figure 3B). The patterns documented the ingestion of mannan and its inclusion into subcellular compartments.

### Interactions of Natural Mannan and Glycoconjugate Formulas 1–8 With Murine Macrophage Cell Line RAW 264.7, and Influence on Cell Proliferation

**Figure 4:**
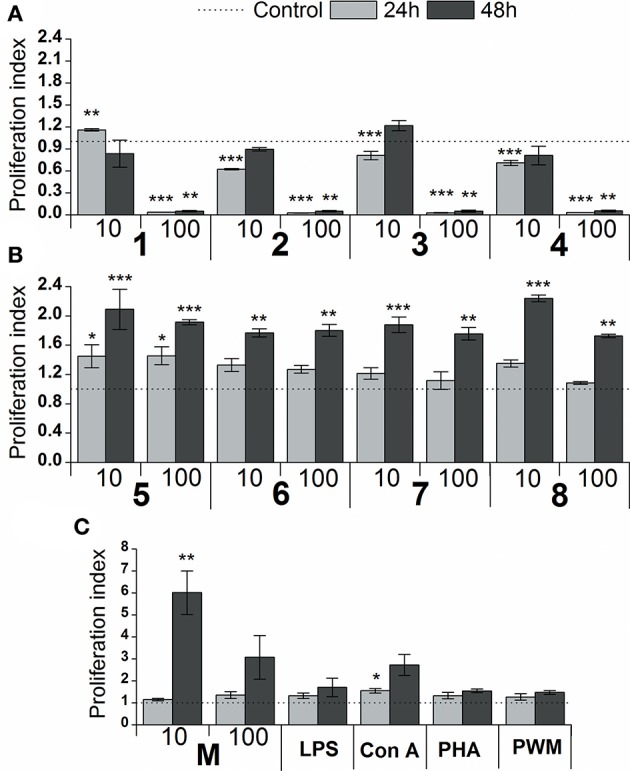
Effect of glycoconjugate formulas **1–8** on proliferation of RAW264.7 macrophages. **(A)**: glycoconjugate formulas **1**, **2**, **3**, and **4**; **(B)**: glycoconjugate formulas **5**, **6**, **7**, and **8**; 10 and 100 μg/mL); **(C)**: *C. albicans* mannan (M, 10 and 100 μg/mL), lipopolysaccharide (LPS, 1 μg/mL), Concanavalin A (Con A, 10 μg/mL), phytohemagglutinin (PHA, 10 μg/mL) and pokeweed mitogen (PWM, 1 μg/mL) for 24 and 48 h. Each value represents the mean ± SD of the proliferation index. The statistical significance of differences between stimulated cells and untreated cells are expressed: ^*^0.01 < *P* < 0.05, ^**^0.001 < *P* < 0.01, ^***^P < 0.001.

The effect of glycoconjugate formulas **1**–**8** on macrophage cell line RAW 264.7 proliferation was monitored by adenosine triphosphate (ATP) bioluminescence as a marker of cell viability (Figure 4). The lower concentration of glycoconjugate formulas **1**–**4** (10 μg/mL, Figure 4A) slightly decreased the proliferation of RAW 264.7 macrophages. Improved proliferation was observed for formula **1**, which is comprised of three α-1,2-Man units (24 h treatment). The higher concentration of formulas **1**–**4** (100 μg/mL, Figure 4A), which are comprised exclusively of α-linkages between Man residues, significantly decreased the proliferation of RAW 264.7 macrophages (between 94 and 98% reduction). As opposed to the α-mannooligosaccharides, treatment of RAW 264.7 macrophages with formulas **5–8**, which also contain β-1,2-linked Man units, slightly increased proliferation after 24 h, and the increase was more significant after 48 h stimulation (Figure 4B). The highest proliferations were observed for the 10 μg/mL concentration of tetramer formula **5**, which contains one terminal β-1,2-linked Man unit (2.1 times higher than control), and hexamer formula **8**, which contains a tetrameric block of β-1,2-linked Man units (2.2 times higher than control). The proliferation of RAW 264.7 macrophages treated by glycoconjugates for 48 h was significantly lower (formulas **1**–**4**: *P* < 0.01, formulas **5–8**: *P* < 0.01) compared with natural *C. albicans* mannan (M, Figure 4C).

### Cytokine Responses of RAW 264.7 Macrophages *in vitro* to Glycoconjugate Formulas 1–8

**Figure 5:**
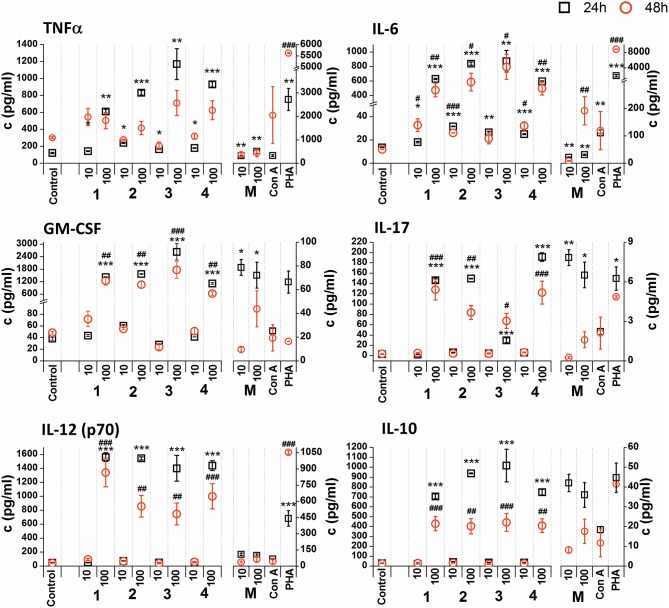
Effect of glycoconjugate formulas **1**–**4** on RAW 264.7 macrophage cytokine production. Negative control represents untreated cells (Control); positive controls: *C. albicans* mannan (M, 10 or 100 μg/mL), Concanavaline A (Con A, 10 μg/mL) and phytohemagglutinin (PHA, 10 μg/mL). All data were normalized to the viable cells' count of untreated control cells of each experiment and are presented as Mean ± SD. Tests were carried out in triplicate. The statistical significance of differences between untreated cells and stimulated cells are expressed: 24 h treatment: ^***^P < 0.001, ^**^0.001 < *P* < 0.01, ^*^0.01 < *P* < 0.05; 48 h treatment: ^*###*^*P* < 0.001, ^*##*^0.001 < *P* < 0.01, ^#^0.01 < *P* < 0.05.

**Figure 6:**
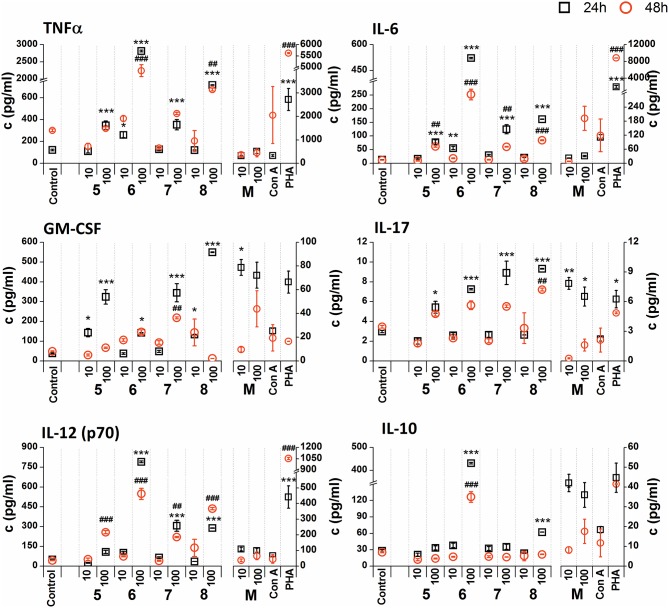
Effect of glycoconjugate formulas **5**–**8** on RAW 264.7 macrophage cytokine production. Negative control represents untreated cells (Control); positive controls: *C. albicans* mannan (M, or 100 μg/mL), Concanavaline A (Con A, 10 μg/mL) and phytohemagglutinin (PHA, 10 μg/mL). All data were normalized to the viable cells' count of untreated control cells of each experiment and are presented as mean ± SD. Tests were carried out in triplicate. The statistical significance of differences between untreated cells and stimulated cells are expressed as follows: 24 h treatment: ^***^P < 0.001, ^**^0.001 < *P* < 0.01, ^*^0.01 < *P* < 0.05; 48 h treatment: ^###^*P* < 0.001, ^##^0.001 < *P* < 0.01, ^#^0.01 < *P* < 0.05.

The *in vitro* stimulatory effect of glycoconjugate formulas **1**–**8** on RAW 264.7 macrophage cytokine production was determined by the levels of pro-inflammatory cytokines TNFα, IL-6, IL-17, IL-12, anti-inflammatory cytokine IL-10, and haemopoietic growth factor GM-CSF in supernatants obtained from cultures of RAW264.7 macrophages after 24 or 48 h treatments [not normalized raw cytokine concentrations (Supplementary Figures 1, 2) and cytokine concentrations normalized to viable cell counts of untreated control RAW 264.7 cells (Figures 5, 6)].

Non-normalized raw cytokine concentrations data showed that stimulation of RAW 264.7 cells with the lower concentration of glycoconjugate formulas **1**–**4** (10 μg/mL), which contain linked Man residues, resulted in a slight increase of TNFα production; maximal effect was observed for formula **1** (24 h treatment: 1.37-fold increase and 48 h treatment: 1.48-fold increase, Supplementary Figure 1). The stimulation of RAW 264.7 macrophages with the higher concentration of glycoconjugate formulas (100 μg/mL) significantly decreased TNFα production (more than 70% decrease compared to the control). However, IL-6 and GM-CSF production showed different concentration dependencies. The higher concentration of glycoconjugate formulas **1**–**4** (100 μg/mL) induced comparable or higher IL-6 and GM-CSF secretion than the lower concentration (10 μg/mL) (Supplementary Figure 1). The highest IL-6 and GM-CSF release was observed for glycoconjugate formula **3** (IL-6: 3.2-fold increase, GM-CSF: 1.9-fold increase).

Glycoconjugate formulas **1**–**4** induced increased IL-17 production (Supplementary Figure 1). The higher concentration of glycoconjugate formulas **1**–**4** (100 μg/mL) induced higher IL-17 secretion, except for glycoconjugate formula **3**, for which IL-17 production declined with increasing glycoconjugate concentration (Supplementary Figure 1). Production of IL-12 showed a structure related dependency (Supplementary Figure 1). The most effective IL-12 inducer was glycoconjugate formula **1**, and induction efficacy declined slightly with increasing number of mannose units in glycoconjugate formulas **1**–**4** (Supplementary Figure 1). Glycoconjugate formulas **1**–**4** did not significantly influence IL-10 production (non-normalized data, Supplementary Figure 1). The results indicated a higher proinflammatory response associated with glycoconjugate formulas **1**–**4**, containing linked Man residues, with significant reduction of RAW 264.7 macrophage proliferation.

The stimulation of RAW 264.7 macrophages with glycoconjugate formulas **5**–**8** showed a different impact on TNFα production compared to glycoconjugate formulas **1**–**4** (Supplementary Figure 2). Higher TNFα production was observed during the shorter exposure period (24 h). The higher tested concentration (100 μg/mL) significantly increased TNFα production, with maximal efficacy for glycoconjugate formula **6** (24 h: 29.4-fold increase, 48 h: 13.4-fold increase compared to the control). Production of IL-6, GM-CSF, IL-17, and IL-12 also showed a concentration dependency, with higher efficacy for the higher concentrations of glycoconjugate formulas **5**–**8** (100 μg/mL). The highest IL-6 secretion was induced by glycoconjugate formula **6** (Supplementary Figure 2, 24 h: 48.8-fold increase, 48 h: 40.0-fold increase, compared to the control). Additionally, glycoconjugate formula **6** induced a strong increase in IL-17, IL-12, and IL-10 production (Supplementary Figure 2). Stimulation with β-mannooligosaccharides **6** and **8** for 24 h markedly increased the production of TNFα (100 μg/mL, *P* < 0.001), IL-6 (100 μg/mL, *P* < 0.001), IL-12 (100 μg/mL, *P* < 0.001), and IL10 (100 μg/mL, *P* < 0.001) compared with natural *C. albicans* mannan.

Due to the tested glycoconjugates having a significant effect on RAW 264.7 macrophage proliferation, especially for glycoconjugate formulas **1**–**4** that contain α-linked Man residues, the raw data of cytokine concentrations in the culture supernatants were normalized to the viable cell counts of untreated control RAW 264.7 cells for each experiment. We observed that the normalization of cytokine concentration data showed no significant trend change for stimulation of RAW 264.7 macrophages with the β-mannooligosaccharide glycoconjugates (formulas **5–8**) (Figure 6). Out of all tested β-mannooligosaccharide glycoconjugates, the most effective cytokine inducers were glycoconjugate formulas **6** and **8**. The highest TNFα (24 h: 23.2-fold increase), IL-6 (24 h, 38.5-fold increase), IL-12 (24 h: 15.6-fold increase), and IL-10 (24 h: 15.3-fold increase) secretion was induced by glycoconjugate formula **6**. The GM-CSF (24 h: 14.5-fold increase) and IL-17 (24 h: 3.1-fold increase) was most effectively induced by β-mannooligosaccharide glycoconjugate formula **8**. Normalization of the cytokine concentration data after stimulation with α-mannooligosaccharide glycoconjugates (formulas **1–4**) (Figure 5) accentuated the release of cytokines induced by the higher concentration of glycoconjugates (100 μg/mL). We observed significant capability to induce TNFα, IL-6, GM-CSF, IL-17, and IL-12 production accompanied by an increase of IL-10 after stimulation with all α-mannooligosaccharide glycoconjugates, induced especially with higher 100 μg/mL concentration, that strongly reduced the proliferation of RAW 264.7 cells. The highest production of TNFα, IL-6, GM-CSF, and IL-10 was observed after the shorter exposure time (24 h) with glycoconjugate formula **3**.

**Figure 7:**
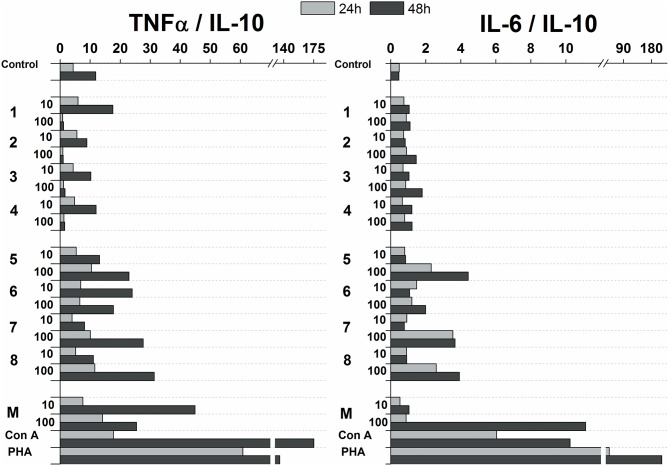
Influence of glycoconjugate formulas **1**–**8** on Th1 and Th2 polarization based on TNFα to IL-10 and IL-6 to IL-10 ratios.

The influence of glycoconjugate formulas **1**–**8** on Th1 and Th2 polarization was revealed based on the TNFα (Th1) to IL-10 (Th2) and IL-6 (Th2) to IL-10 (Th2) ratios (Figure 7). Th1 dominance was represented by a higher ratio, while a lower ratio expressed a Th2 dominated environment. Concerning the ratios following 24 and 48 h exposures with 100 and 10 μg/mL of glycoconjugate formulas **1**–**4**, Th1 dominance based on the TNFα/IL-10 ratio was revealed for conjugate formulas **1** and **4**, while conjugate formulas **2** and **3** exerted Th1 dominance with higher TNFα/IL-10 ratios over IL-6/IL-10 ratios only at the lower concentration (10 μg/mL) after 48 h exposure. For conjugate formulas **5**–**8**, the values of the TNFα/IL-10 ratios overcame the values of the IL-6/IL-10 ratios following 24 and 48 h exposures with both concentrations for all conjugates, reflecting Th1 dominance.

The resulting *in vitro* proinflammatory effect of glycoconjugate formulas **5-8**, containing terminal β-mannosyls, overcame that of the α-mannooligosaccharides. This was supported by statistically insignificant correlations between the release of proinflammatory cytokines following 24 and 48 h exposures with α-mannooligosaccharides. Significant overall correlations were determined between the release of proinflammatory cytokines induced by individual β-mannooligosaccharides glycoconjugate formulas following 24 h exposure: TNFα and IL-6 (*R* = 0.994 *p* = 5.38 ×10^−7^), TNFα and IL-12 (*R* = 0.969 *p* = 6.71 ×10^−5^), and IL-12 and IL-6 (*R* = 0.989 *p* = 2.61 ×10^−6^). After 48 h, a significant correlation was also revealed between IL-17 and IL-12 (*R* = 0.877 *p* = 0.0042).

## Discussion

Several attempts have been made to synthesize relevant mannan epitopes with immunobiological effectiveness. Synthetically prepared mannooligosaccharides mimicking *Candida* antigenic factors (Karelin et al., 2010, 2015, 2016, 2017) represent promising study models to establish the immunomodulating activity of such formulas on humoral and cellular immunity for subcellular anti-*Candida* vaccine construction (Paulovicova et al., 2010, 2012, 2014; Paulovicova L. et al., 2013).

The immunobiological importance and vaccination potency of synthetically prepared β-1,2-mannopyranosyl trisaccharide mimicking the structure of the *C. albicans* cell surface epitope has previously been studied (Xin et al., 2008, 2012; Costello and Bundle, 2012; Cartmell et al., 2015; Bundle et al., 2018). Next, a novel tetrasaccharide construct consisting of β-1,2-mannopyranosyl trisaccharide and α-mannopyranoside was designed and suggested as a model of the *C. albicans* phosphodiester epitope (Dang et al., 2012). Glycoarrays formed by biotinylated oligosaccharides loaded on streptavidin-coated surfaces were previously shown to be indispensable instruments for the investigation of carbohydrate antigen recognition by immune cells (Komarova et al., 2015, 2018; Akhmatova et al., 2016; Paulovicova et al., 2016, 2017; Kurbatova et al., 2017; Argunov et al., 2019; Schubert et al., 2019).

Moreover, the sera reactivity and determination of antigen-specific isotypic antibodies against synthetically prepared mannooligosaccharides were evaluated in a cohort of patients with vulvovaginal candidosis (Karelin et al., 2016; Paulovicova et al., 2016, 2017). Postvaccination antisynthetic heptamannoside polyclonal sera inhibited growth of the azole-resistant clinical strain *C. albicans* CCY 29-3-164 and reduced the number of colony-forming units throughout an experimental mucosal infection (Paulovicova E. et al., 2013). Next, sera cytokine patterns of Th1/Th2/Th17 polarization of immune responses by synthetic oligosaccharide–BSA conjugates revealed a tight structure-activity relationship (Paulovicova E. et al., 2013; Paulovicova et al., 2017).

Here, the proliferating and cytokine-inducing activities of a series of synthetically prepared nanopolymers mimicking native *C. albicans* cell wall immunogenic moieties were studied using RAW264.7 cell exposure (Figure 4). The cell proliferation results revealed almost immunoinhibitory activity of α- mannoside formulas **1**–**4** (trisaccharide through hexasaccharide), with more pronounced activity with increasing concentration (*P* < 0.001), in contrast with native *C. albicans* cell wall mannan (Figure 4). These findings concur with previously published studies (Podzorski et al., 1989, 1990) that reported the immunoinhibitory influence of members of a family of mannose oligosaccharides (disaccharide through hexasaccharide) derived from cetyltrimethylammonium bromide (CTAB) mannan (native *C. albicans* mannan prepared by complexation with CTAB). CTAB mannan was a potent stimulator of lymphoproliferative when added to human peripheral blood mononuclear cells (PMBCs) from donors responsive to *Candida*; it had no inhibitory influence on lymphoproliferation induced by *Candida* or other antigens. Two major oligomannosyl components, mannobiose and mannotriose, of CTAB mannan with an inhibitory effect on cell proliferation were demonstrated to be bound mainly through α(1,2) linkages (Hayette et al., 1992). In contrast, synthetically prepared mannooligosaccharide formulas **5–8** with terminal β-mannosyl units (Figure 3) exerted a stimulatory effect on RAW264.7 cell proliferation (*P* < 0.001). Thus, the immunobiological properties of the studied mannooligosaccharides are dose- and structure-dependent. Cell release of interleukins and growth factors associated with inflammation and proliferation was induced by mannooligomers to different extents depending on the oligomer structures (normalized data: Figures 5, 6, not normalized data: Supplementary Figures 1, 2).

Upregulation of cytokines such as TNFα, IL-6, IL-12, GM-CSF was more evident with mannooligosaccharides with terminal β-mannosyl units. Acceleration of secretion of anti-inflammatory cytokine IL-10 with Th1-inhibiting properties was also revealed with β-mannooligosaccharides (Supplementary Figure 2). Association between pro- and anti-inflammatory cytokines, in addition to Th1, Th2 and Th17 polarization, is an important prerequisite for the assessment of immunogenic substance behavior. The influence of glycoconjugate formulas **1**–**8** on Th1 and Th2 polarization, based on TNFα to IL-10 and IL-6 to IL-10 ratios (Figure 7), resulted in a predominant Th1 immune response. Th1 dominance, represented by higher TNFα/IL-10 and IL-6/IL-10 ratios with higher dose and following prolonged treatment, was revealed with tested **1**–**8** formulas, and was more evident for formulas **5**–**8** with β-terminal mannosyls; the Th2 dominated environment was not determined. The observed immune response was tightly associated with dose, exposure time, and selected signature cytokines. The TNFα/IL-10 ratio was more descriptive than the IL-6/IL-10 ratio, presumably due to dual IL-6 roles, i.e., anti-inflammatory activities of IL-6 are mediated by classic signaling, whereas proinflammatory responses of IL-6 are mediated by trans-signaling (Scheller et al., 2011). Saijo et al. reported induced release of cytokines, such as IL-12p40, IL-6, TNFα, and IL-10, from wild-type bone marrow-derived dendritic cells (BMDCs) by treatment with *C. albicans* water soluble fraction (CAWs) and *C. albicans* mannans (Saijo et al., 2010). Moreover, they also observed the secretion of yeast- and hyphae-specific cytokines following cell exposure with both *C. albicans* morphoforms. *C. albicans* mannan, glucomannoprotein and phospholipomannan, containing β-1,2 oligomannosides, induced TNFα in association with degree of polymerization (DP). Jouault et al. noted that TNFα-release occurred most in the presence of relatively long chains of β-oligomannosides (i.e., an oligomannoside comprised of eight mannose units was superior to shorter chains, and oligomannosides of less than four mannose units were not active) (Jouault et al., 1995). Evidently a minimal DP of 4 was necessary to induce production of cytokine (Poulain et al., 1997). With synthetically prepared α- and β- oligomannosides, effective TNFα release was triggered by trimannosides. The ability of β-oligomannosides to induce release of TNFα was also demonstrated by Cutler (2001).

Additionally, cell exposure to conjugate formula **6**, which comprises 2 β and 3α mannosyls, exerted the highest media release of IL-12p70, IL-6, TNFα, and IL-10 (Figure 6). Interestingly, conjugate formula **8**, with 4β and 2α-linked mannosyls, induced higher IL-17 and regulatory GM-CSF cell release than the other β-oligomannosides (Figure 6).

## Conclusions

Our data suggest an immunobiological role for synthesized mannooligosaccharides that closely resemble *Candida* cell wall mannooligomers. The observed Th1/Th2/Th17 immune responses were tightly associated with structure, dose, exposure time, and selected signature cytokines. Glycoconjugate formulas **5**–**8**, with terminal β-mannosyl-units, tended to be more potent than glycoconjugate formulas **1**–**4** in terms of *Candida* relevant cytokines IL-12 p70, IL-17, GM-CSF, IL-6, and TNFα induction and cell proliferation, and this tendency was associated with structural differences between the studied glycoconjugate formulas. Obtained results warrant further systematic investigation of the immunological properties of carbohydrate antigens of the *Candida* cell wall toward the selection of efficient structures suitable for application as immunomodulative agents either for *in vitro Candida* diagnostics or prospectively for subcellular anti-*Candida* vaccine design.

## Data Availability Statement

All datasets generated for this study are included in the article/Supplementary Material.

## Author Contributions

EP, LP, and NN contributed to the conception and design of the study, performed the immunobiological research and analyzed data, acquired funding, and prepared the original draft. PF performed the modification and characterization of mannan. AK, YT, and VK performed the chemical syntheses and analyzed data. All authors contributed to manuscript revision, read, and approved the submitted version.

### Conflict of Interest

The authors declare that the research was conducted in the absence of any commercial or financial relationships that could be construed as a potential conflict of interest.
