# Supplementary material for: Importance of Candida Antigenic Factors: Structure-Driven Immunomodulation Properties of Synthetically Prepared Mannooligosaccharides in RAW264.7 Macrophages
Source: Front Cell Infect Microbiol. 2019 Nov 8;9:378. doi: 10.3389/fcimb.2019.00378 (PMC6856089; doi:10.3389/fcimb.2019.00378)
Supplement: Supplementary file 1 [file Table_1.DOCX]

Supplementary Material

- 1. **Supplementary Figures**

**Supplementary Figure 1. │** Effect of glycoconjugates **1**-**4** on RAW 264.7 macrophages cytokines production.

Not normalized raw data of cytokines’ concentrations (pg/ml) in media after stimulation of RAW264.7 macrophages (24 h or 48 h) in response to stimulation with 10 µg/ml or 100 µg/ml concentration of glycoconjugates **1**-**4**; negative control represents untreated cells (Control); *C. albicans* mannn (M, 10 µg/ml or 100 µg/ml), Concanavaline A (Con A, 10 µg/ml) and phytohemagglutinin (PHA, 10 µg/ml) were used as positive controls. All data are presented as Mean ± SD . Tests were carried out in triplicate. The statistical significance of differences between untreated cells and stimulated cells are expressed; 24h treatment: *** – P<0.001, ** – 0.001<P<0.01, * – 0.01<P<0.05, 48h treatment: ### – P<0.001, ## – 0.001<P<0.01, # – 0.01<P<0.05.

**Supplementary Figure 2. │** Effect of glycoconjugates **5**-**8** on RAW 264.7 macrophages cytokines production.

Not normalized raw data of cytokines’ concentrations (pg/ml) in media after stimulation of RAW264.7 macrophages (24h or 48h) in response to stimulation with 10 µg/mL or 100 µg/mL concentration of glycoconjuates **5**-**8**; negative control represents untreated cells (Control); *C. albicans* mannn (M, 10 µg/ml or 100 µg/ml), Concanavaline A (Con A, 10 µg/ml) and phytohemagglutinin (PHA, 10 µg/ml) were used as positive controls. All data are presented as Mean ± SD. Tests were carried out in triplicate. The statistical significance of differences between untreated cells and stimulated cells are expressed; 24h treatment: *** – P<0.001, ** – 0.001<P<0.01, * – 0.01<P<0.05, 48h treatment: ### – P<0.001, ## – 0.001<P<0.01, # – 0.01<P<0.05.
